# Knowledge and Misconceptions About Parkinson’s Disease in Lebanon: A Cross‐Sectional Survey

**DOI:** 10.1155/padi/1010419

**Published:** 2026-07-08

**Authors:** Noura El-Loubani, Hanan Hijazi, Sarah Mawed, Layal El Bayda, Rabih Roufayel, Hervé Kovacic, Ziad Fajloun, Jeanne d’arc Bacha

**Affiliations:** ^1^ Faculty of Public Health, Jinan University, Tripoli, 900, Lebanon, jnu.edu.cn; ^2^ Faculty of Sciences, Jinan University, Tripoli, 900, Lebanon, jnu.edu.cn; ^3^ College of Engineering and Technology, American University of the Middle East, Egaila, 54200, Kuwait, aum.edu.kw; ^4^ Aix-Marseille Univ, CNRS, INP, Inst Neurophysiopathologie, Marseille, 13385, France, univ-amu.fr; ^5^ Department of Cell Culture, Laboratory of Applied Biotechnology (LBA3B), Azm Center for Research in Biotechnology and its Applications, EDST, Lebanese University, Tripoli, 1300, Lebanon, ul.edu.lb; ^6^ Department of Biology, Faculty of Sciences 3, Lebanese University, Campus Michel Slayman Ras Maska, Tripoli, 1352, Lebanon, ul.edu.lb

**Keywords:** cross-sectional study, healthcare sector, Lebanese population, Lebanon, neurodegenerative disorder, Parkinson’s disease

## Abstract

**Background:**

Parkinson’s disease (PD) is a neurodegenerative disorder affecting brain structure and function. It is manifested by unintended movements, characterized by tremors, stiffness, and difficulty with balance and coordination. According to the statistics reported by the World Health Organization (WHO), the prevalence of PD has doubled over the past 25 years. Nowadays, PD has become a major global health burden, including in the Middle East, particularly in Lebanon. Since 2000, the number of people living with PD has increased by 81%, whereas mortality associated with the disease has risen by more than 100%.

**Objectives:**

Our goal in this study is to assess the level of knowledge and awareness of PD among community‐dwelling Lebanese individuals across different age groups and from various segments of society.

**Methods:**

We conducted a cross‐sectional survey of the Lebanese population, encompassing individuals from various regions of the country. A questionnaire covering participants’ sociodemographic characteristics, as well as the etiology, symptoms, management, and other aspects of PD, was distributed via Google Form to individuals from diverse professional, educational, and socioeconomic backgrounds. Using a systematic sampling method, we gathered data from 582 participants.

**Results:**

Among the 582 participants, we observed significantly lower levels of knowledge regarding the symptoms, causes, and treatments of PD, particularly among individuals employed outside the healthcare sector and those with lower levels of education.

**Conclusion:**

The outcomes showed us that there is an extensive knowledge gap in neurological disorders, especially in PD, concerning the etiologies of its occurrence and how to deal with patients having the disorder. Our community in Lebanon needs more awareness about PD, which will play an important role in the acceptance and understanding of the disorder.

## 1. Introduction

Parkinson’s disease (PD) is a chronic progressive neurodegenerative disorder characterized by early prominent death of dopaminergic neurons and iron overload in the substantia nigra pars compacta (SNpc) of the midbrain [1].

Dopamine levels are reduced in the basal ganglia as a result of degeneration of the SNpc in PD [2]. This dopamine deficiency leads to classical Parkinsonian motor symptoms, including bradykinesia, tremor, rigidity, and postural instability [3]. PD is equally associated with nonmotor features via neuropsychiatric dysfunction (mood disorders, dementia, and apathy), sleep disorders, autonomic dysfunction (orthostatic hypotension [OH], urogenital dysfunction, and constipation), sensory symptoms (olfactory dysfunction), and pain [4]. PD is a challenging neurological disorder, as its symptoms can significantly impact daily life and are often difficult to manage [5]. Moreover, diagnosis can be uncertain, especially in the early stages when clinical manifestations are subtle and nonspecific. Additionally, two other disorders share similar patterns of clinical deficits in the early stages with those of PD. These diseases are Steele–Richardson–Olszewski syndrome and Shy–Drager syndrome, which are characterized by Parkinsonian features such as tremors, rigidity, and bradykinesia [6]. However, these disorders have distinguishing features but fail to appear earlier.

According to the statistical studies established by the World Health Organization (WHO), the prevalence of PD has doubled in the past 25 years, and over 8.5 million people worldwide were living with PD in 2019. WHO estimated that PD surged 81% since 2000 and caused 329,000 deaths. Therefore, attention has been drawn to the growing burden of PD in the Middle East and North Africa (MENA) region. To assess this burden, researchers retrieved between 1990 and 2019 the prevalence, mortality, and disability‐adjusted life years (DALYs) attributable to PD across 21 countries of the MENA region [7]. They found that the prevalence, the death rate, and the DALY rate of PD have risen between 1990 and 2019 by 15.4%, 2.3%, and 0.9%, respectively [7].

In Lebanon, PD has shown an increasing trend in epidemiological parameters between 1990 and 2019, with prevalence, mortality, and DALYs rising by 9.3%, 14.1%, and 14.2%, respectively [7]. Despite this increase, these figures remain lower in Lebanon compared to neighboring countries such as Qatar, the United Arab Emirates, and Oman [7]. One possible explanation is the cultural stigma associated with PD and other chronic illnesses, which is prevalent in the Middle East, North Africa, and South Asia (MENASA) region [8]. This stigma may prevent individuals from seeking medical attention, contributing to delayed diagnosis and underreporting [9].

Given the relatively low reported prevalence, it is plausible that public knowledge and awareness of PD in Lebanon are limited. Notably, there is a lack of studies comprehensively evaluating the public’s understanding of the disease. A recent study by Abramian [10] validated scales forassessing knowledge and attitudes toward PD among the Lebanese population;factors associated with awareness were identified and proposed strategiesto support public health campaigns. However, this study did not delve deeply into awareness of specific aspects such as risk factors, treatment options, or the influence of educational background and occupational sector on PD knowledge. Similarly, research conducted in Saudi Arabia revealed a general lack of awareness regarding PD symptoms and causes, highlighting the broader regional need for educational interventions to address misconceptions and improve public understanding [11].

Given the cumulative percentage of PD in Lebanon, particularly over the past three years, and the limited research on public awareness of the disease, we conducted a survey targeting a diverse cross‐section of the population. This study aimed to evaluate the level of knowledge and awareness regarding PD among Lebanese individuals from various educational backgrounds, occupational sectors, and social groups.

## 2. Materials and Methods

### 2.1. The Study Design

A cross‐sectional study was conducted among 582 participants from the Lebanese population to assess awareness of PD. The survey was developed using Google Forms and disseminated electronically through widely used social media platforms, including WhatsApp, Facebook, Instagram, and Twitter. The survey invitation included a brief explanation of the study objectives and encouraged voluntary participation. The questionnaire link (https://docs.google.com/forms/d/e/1FAIpQLSexS9Ni7EgJQLqrmkmepY8UDMZV2S4S8OpZxONBcve7B21yaQ/vieform?vc=0%26c=0%26w=1%26flr=0) was shared across different Lebanese regions to maximize outreach among individuals from diverse educational, occupational, and social backgrounds.

Participants were recruited using a convenience self‐selection sampling method, whereby eligible individuals voluntarily completed the online questionnaire. Data collection was conducted between November 2022 and July 2023.

Due to the open online dissemination strategy, the exact response rate could not be accurately determined. Responses were screened for completeness and consistency before inclusion in the final analysis. Questionnaires containing substantial missing or incomplete data were excluded from the study.

### 2.2. Participant Recruitment

Participants were recruited from the general Lebanese population using a convenience self‐selection sampling approach. Eligible participants included men and women aged 15 years or older who were able to understand and voluntarily respond to the survey questionnaire. Exclusion criteria included non‐Lebanese expatriates, individuals younger than 15 years of age, and incomplete questionnaire submissions. Participation in the study was entirely voluntary, and no financial incentives were provided.

### 2.3. Questionnaire

The survey instrument was developed based on an extensive review of previously published literature related to PD awareness and knowledge assessment. In addition, knowledge items were developed based on studies and current evidence available at the time of questionnaire design; however, some topics represent evolving areas of research.

The questionnaire was structured into three main sections to comprehensively evaluate participants’ awareness and knowledge of PD. The first section gathered sociodemographic characteristics, including age, gender, educational attainment, and occupation type. The second section assessed participants’ prior exposure to PD, including whether they had previously heard of the disease, had a family history of PD, or had known or encountered individuals diagnosed with the condition. The third section consisted of knowledge‐based questions evaluating participants’ understanding of PD prevalence, etiology, symptoms, and available treatment approaches.

To ensure content validity, the initial version of the questionnaire was reviewed by specialists in neurology and public health, who evaluated each item for its clarity, relevance, comprehensiveness, and alignment with the study objectives. Based on their recommendations, minor modifications in wording and question structure were implemented to improve clarity and reduce ambiguity.

The validation process followed a two‐phase approach consisting of expert evaluation and pilot testing. The revised questionnaire was subsequently administered to a pilot sample of 20 individuals representative of the target population to assess readability, comprehension, and completion time. Feedback obtained during the pilot phase identified minor linguistic and structural issues, which were corrected before the final distribution of the survey. Participants involved in the pilot study were excluded from the final statistical analysis.

The questionnaire was administered in English/Arabic/both languages. For the Arabic version, a forward–backward translation procedure was performed by bilingual experts to ensure linguistic accuracy and conceptual equivalence between both versions. Linguistic validation was further confirmed during the pilot testing phase.

The final version of the questionnaire was distributed electronically through online platforms, and a copy of the questionnaire is provided as Supporting Information (Supporting File 1).

### 2.4. Sample Size Calculation and Data Quality Control

The minimum required sample size was calculated using the single population proportion formula:

(1) n=Z2P1−Pd2,

where *n* represents the required sample size, *Z* corresponds to the standard normal distribution value at a 95% confidence level (1.96), P represents the estimated response distribution set at 50% due to the absence of prior national data, and *d* represents the margin of error set at 5%.

The sample size was calculated using the online sample size calculator Raosoft, based on the Lebanese population size reported by the World Bank Open Data.

The minimum required sample size was determined to be 385 participants. To compensate for potential nonresponse and incomplete questionnaires, additional responses were targeted. The final sample included 582 participants, exceeding the minimum required sample size. Statistical analysis was conducted with a significance level of 0.05 and an estimated statistical power of 80%.

Several procedures were implemented to ensure data quality. The questionnaire was reviewed prior to dissemination to ensure clarity and comprehension. Responses were screened for completeness, consistency, and duplicate entries before statistical analysis. Data cleaning procedures were conducted to minimize errors and improve the reliability of the collected data.

### 2.5. Data Analysis

All data analyses were performed using IBM SPSS Statistics Version 25. Categorical data were presented as frequencies and percentages. Nominal variables were analyzed using the chi‐square test or Fisher’s exact test when more than 20% of the expected cell counts were less than 5. Ordinal variables, as well as comparisons between nominal and ordinal variables, were assessed using the Kruskal–Wallis test. A *p*‐value of less than 0.05 was considered statistically significant (^∗^indicates *p* < 0.05; ^∗∗^indicates *p* < 0.01, denoting a highly significant association).

### 2.6. Ethical Considerations

The study protocol and survey instrument were reviewed and approved by the Ethics Review Board (ERB) of the Faculty of Public Health at Jinan University (ERB‐FPH‐012022). Following ethical approval, the survey was disseminated through social media to various segments of the Lebanese population.

All participants were provided with detailed information about the purpose and nature of the study before participation. Informed consent was obtained electronically at the beginning of the survey. Participants were required to acknowledge their voluntary participation by selecting a consent confirmation option before accessing the questionnaire.

Participants aged 15 years or older were eligible for inclusion. For participants under the age of 18 years, parental or guardian consent was obtained in addition to participant assent.

The study was conducted using an online self‐administered questionnaire. Data security was ensured, and technical measures were implemented to minimize duplicate responses. Confidentiality and anonymity were ensured, and no personally identifiable information was collected. Participation was entirely voluntary, and respondents could withdraw from the survey at any time without any consequences.

## 3. Results

### 3.1. Analysis of Sociodemographic Distributions for Responders

The demographic characteristics of the study participants reveal a predominantly young and educated sample, with the majority aged between 20 and 30 years (57.4%) and over half (58.6%) holding undergraduate degrees. A significant portion of respondents (81.6%) were female, indicating a gender imbalance that may influence knowledge and awareness outcomes. Most participants were unemployed (50.7%), likely reflecting a large student population, while others were employed in the educational (13.4%) and healthcare (11.5%) sectors (Table 1). The high educational attainment and presence of respondents from relevant professional fields suggest a population that may possess a foundational understanding of health‐related issues, potentially influencing the accuracy and depth of their responses in the study context.

**TABLE 1:** Demographic characteristics of the participants.

Characteristics	*n* (%)
Age range in years (*n* = 573)	
[15–20]	83 (14.50)
[20–30]	329 (57.40)
[31–40]	90 (15.70)
[41–50]	44 (7.60)
> 50	27 (4.70)
Gender (*n* = 582)	
Male	107 (18.40)
Female	475 (81.60)
Education (*n* = 582)	
Intermediate education	17 (2.90)
Secondary education	52 (9.00)
Undergraduates	341 (58.60)
Master’s degree	148 (25.50)
PhD degree	24 (4.00)
Occupation (*n* = 582)	
Unemployed	295 (50.70)
Employee	142 (24.40)
Educational sector	78 (13.40)
Healthcare sector	67 (11.50)
First aid crew	2 (0.30)
Nurse	36 (6.20)
Doctor	8 (1.40)

### 3.2. Assessment of Participants’ Knowledge and Awareness of PD

The findings reveal a relatively moderate level of general awareness regarding PD among participants, with 73.4% reporting that they had heard of PD. Despite this, personal experience with the disease was limited, as only 0.7% reported having PD and 9.1% had a family member with the condition. Furthermore, 39.3% had ever met someone with PD, indicating limited direct exposure.

Regarding disease understanding, only 4.1% of respondents believed that PD can be completely cured, whereas 43% were uncertain, highlighting substantial gaps in knowledge and awareness of the disease. The vast majority of participants (97.8%) perceived that awareness of PD in Lebanon is insufficient.

Concerning the potential association between COVID‐19 and PD, 29.9% of participants believed a link exists, while 8.6% were uncertain, reflecting variability in perceptions and limited clarity regarding emerging scientific evidence (Table 2).

**TABLE 2:** The participants’ knowledge and awareness of Parkinson’s disease and related aspects (*n* = 582).

Question	Yes *n* (%)	No *n* (%)	I don’t know *n* (%)
Have you ever heard of PD?	427 (73.40)	155 (26.60)	0 (0.0)
Do you have PD?	4 (0.70)	578 (99.30)	0 (0.0)
Do you have a family member with PD?	53 (9.10)	478 (82.10)	51 (8.80)
Have you ever met a patient with PD?	229 (39.30)	353 (60.70)	0 (0.0)
Can Parkinson’s disease be completely cured?	24 (4.10)	308 (52.90)	250 (43.00)
Is the level of awareness regarding PD in Lebanon sufficient?	13 (2.20)	569 (97.80)	0 (0.0)
Can COVID‐19 virus cause PD?	174 (29.90%)	358 (61.50%)	50 (8.60%)

Overall, these findings highlight the need for strengthened public education and awareness initiatives on PD in Lebanon.

### 3.3. Participants’ Awareness of PD Epidemiology and General Characteristics

The analysis of participants’ responses regarding epidemiological aspects of PD reveals variability in awareness across several domains. When asked about the perceived frequency of PD in Lebanon, only 29.9% selected the 5%–10% range. Regarding the age of onset, 37.3% of respondents reported that PD typically begins after the age of 60 years, whereas 45.6% believed that there is no specific age of onset, indicating variability in perceptions of the disease’s demographic profile.

A higher level of awareness was observed regarding gender distribution, as 76.5% of participants reported that men are more likely to develop PD. However, responses varied regarding genetic contribution to PD. While 37.3% of respondents selected the 10%–15% range, a substantial proportion overestimated the genetic contribution to PD, with 30.9% choosing 50%–60% and 8.6% selecting 90% (Table 3).

**TABLE 3:** Participants’ perceptions of epidemiological characteristics of Parkinson’s disease (*n* = 582).

Questions and answers	Responses *n* (%)
What do you think is the frequency of Parkinson’s disease in Lebanon?	
0.5%–1%	119 (20.60%)
1%–5%	217 (37.30%)
5%–10%^†^	174 (29.90%)
> 10%	71 (12.20%)
At what age do you think Parkinson’s disease typically appears?	
No specific age	265 (45.60%)
< 40 years old	45 (7.70%)
> 40 years old	163 (28.10%)
> 60 years old^†^	218 (37.30%)
Who do you think is more likely to develop Parkinson’s disease?	
Men^†^	445 (76.50%)
Women	137 (23.50%)
What is your perception of the contribution of genetic factors to Parkinson’s disease?	
0%–10%	135 (23.20%)
10%–15%^†^	217 (37.30%)
50%–60%	180 (30.90%)
90%	50 (8.60%)

^†^Items reflect participants’ perceptions of Parkinson’s disease epidemiology and genetics and are based on evidence from the literature.

Overall, these findings highlight variability in participants’ awareness of PD epidemiology and emphasize the need for targeted educational initiatives to improve public understanding, particularly regarding disease prevalence, age of onset, and genetic contribution.

### 3.4. Participants’ Perceptions of Etiology and Risk Factors of PD

Participants demonstrated varied levels of awareness regarding nutritional factors potentially associated with PD. Approximately 67.9% of participants identified vitamin B12 deficiency as a factor that has been discussed in relation to PD in previous studies, whereas 37.5% identified vitamin B9 deficiency. In addition, 39.2% and 9.6% of respondents selected vitamin D and vitamin C deficiencies, respectively, reflecting variability in participants’ understanding of nutritional factors potentially associated with PD (Table 4).

**TABLE 4:** Participants’ perceptions of nutritional factors associated with Parkinson’s disease (*n* = 582).

Questions and answers	Responses *n* (%)
Which of the following vitamin deficiencies have been reported in some studies to be associated with PD?	
Vitamin B12^†^	394 (67.90%)
Vitamin B9^†^	218 (37.50%)
Vitamin D	228 (39.20%)
Vitamin C	56 (9.60%)
Which dietary patterns or nutritional factors have been investigated in relation to PD risk?	
Meat‐based^†^	261 (44.80%)
Rich in dairy products^†^	146 (25.10%)
Meat and plant‐based	122 (21.00%)
Vegetarian	192 (33.00%)

^†^Factors reported in previous studies as potentially associated with Parkinson’s disease; evidence remains limited or inconclusive for some associations.

Regarding dietary patterns, 44.8% of participants identified meat‐based diets as potentially associated with PD risk, whereas 25.1% selected diets rich in dairy products, 21% selected mixed meat‐ and plant‐based diets, and 33% selected vegetarian diets (Table 4). These findings indicate differing perceptions among participants regarding the possible relationship between nutrition and PD. Given that evidence regarding several nutritional and dietary associations with PD remains evolving and, in some cases, inconclusive, these results should be interpreted cautiously. Nevertheless, the findings highlight the importance of improving public education regarding evidence‐based nutritional factors and neurodegenerative diseases.

The data in Table 5 reveal varying levels of awareness among participants regarding factors associated with PD. Among commonly reported risk factors, genetic predisposition was the most frequently selected (67.2%), followed by aging (57%) and vitamin deficiency (48.3%).

**TABLE 5:** Participants’ awareness of risk factors associated with Parkinson’s disease (*n* = 582).

Question and answers	Responses *n* (%)
Which of the following factors have been reported or investigated in association with Parkinson’s disease (PD)?	
Low level of dopamine^†^	197 (33.80%)
Getting old^†^	332 (57.00%)
Chronic diseases^†^	124 (21.30%)
Genetic factors^†^	391 (67.20%)
Head trauma^†^	177 (30.40%)
Diabetes^†^	42 (7.20%)
Epilepsy^†^	106 (18.20%)
Vitamin deficiency^†^	281 (48.30%)
Milk and dairy products^†^	73 (12.50%)
Stress and fear	170 (29.20%)
Influenza	37 (6.40%)
Smoking	108 (18.6%)
Brain tumors	142 (24.40%)
Brain stroke	95 (16.30%)
Mental pressure	161 (27.70%)
Sadness or depression	159 (27.30%)

^†^Factors reported in previous studies as potentially associated with Parkinson’s disease; evidence remains limited or inconclusive for some associations.

A considerable proportion of participants identified low dopamine levels (33.8%), reflecting awareness of a key pathophysiological feature of PD rather than a risk factor, indicating variability in understanding of the disease’s biological mechanisms.

Recognition of other established or discussed risk factors, such as chronic diseases (21.3%), head trauma (30.4%), diabetes (7.2%), and epilepsy (18.2%), remained relatively low, suggesting limited awareness of several medically described associations.

In addition, some participants selected non‐established or weakly supported factors, including stress and fear (29.2%), mental pressure (27.7%), sadness or depression (27.3%), and smoking (18.6%), reflecting variability in perceived risk attribution.

Overall, these findings highlight heterogeneous perceptions of PD risk factors and emphasize the need for targeted educational interventions to improve public understanding of the disease’s established risk profile.

### 3.5. Participants’ Perceptions of Clinical Manifestations of PD

The analysis of participants’ awareness of PD symptoms indicates varying levels of recognition across both motor and nonmotor manifestations. Notably, rest tremor was identified by all participants (100%), suggesting a high level of public familiarity with this hallmark motor symptom of PD. Other motor‐related symptoms, such as gait dysfunction (72.5%), slow movement (63.6%), walking difficulty (60%), and muscle stiffness (51%), were also widely recognized, although awareness progressively decreased for less specific manifestations.

In contrast, awareness of nonmotor symptoms was substantially lower. Sleep disturbances were identified by 41.4% of participants, depression by 39.9%, and constipation by 23.4%, despite their well‐established clinical relevance in PD. Masked face, a subtle motor feature of the disease, was identified by 33.7% of respondents.

Additionally, a proportion of participants selected symptoms not typically associated with PD, such as hypertension (11.9%), anorexia (18.6%), and hypoxia (15.5%), indicating variability in participants’ understanding of the disease’s clinical presentation (Table 6). Overall, these findings suggest greater awareness of classical motor symptoms compared with nonmotor features, highlighting the need for improved public education regarding the full clinical spectrum of PD.

**TABLE 6:** Awareness of Parkinson’s disease symptoms among participants (*n* = 582).

Question and answers	Responses *n* (%)
Which of the following symptoms have been reported or are commonly associated with Parkinson’s disease (PD)?	
Rest tremor^†^	582 (100%)
Walking difficulty^†^	349 (60.00%)
Gait dysfunction^†^	422 (72.50%)
Muscle stiffness^†^	297 (51.00%)
Behavioral changes^†^	175 (30.10%)
Slow movement^†^	370 (63.60%)
Masked face^†^	196 (33.70%)
Constipation^†^	136 (23.40%)
Depression^†^	232 (39.9%)
Difficulty in sleeping^†^	241 (41.40%)
Hypertension	69 (11.90%)
Anorexia	108 (18.60%)
Hypoxia	90 (15.50%)
Dementia	152 (26.10%)

^†^Symptoms reported in the literature as being associated with Parkinson’s disease and included to assess participants’ awareness, recognition, and interpretation of clinical manifestations may vary among individuals.

### 3.6. Participants’ Perceptions of PD Management Strategies

The data presented in Table 7 indicate a generally good level of awareness among participants regarding approaches used in the management of PD, although variations in understanding were observed. The majority of participants identified medication as a commonly used management strategy (79.9%), while more than half reported exercise (56%), physiotherapy (50.9%), and vitamin supplements (56.5%) as relevant approaches. Awareness of deep‐brain stimulation (DBS) surgery, an advanced therapeutic option for PD, was comparatively lower (18%), suggesting limited familiarity with surgical interventions.

**TABLE 7:** Participants’ awareness of strategies for managing Parkinson’s disease symptoms (*n* = 582).

Question and answers	Responses *n* (%)
Which of the following approaches are you aware of as being used to manage PD symptoms?	
Medicine^†^	465 (79.90%)
DBS surgery^†^	105 (18.00%)
Exercise^†^	326 (56.00%)
Nicotine/smoking	14 (2.40%)
Physiotherapy^†^	296 (50.90%)
Healthy diet	289 (49.80%)
Vitamin supplements^†^	329 (56.50%)
Caffeine consumption	36 (6.20%)
Alcohol consumption	10 (1.70%)
Social support	133 (22.90%)
Psychiatric/psychological therapy	212 (36.40%)

^†^Evidence‐based therapeutic approaches for the management of Parkinson’s disease symptoms, as reported in clinical literature; participants’ responses reflect awareness rather than clinical correctness.

Adjunctive and supportive approaches such as a healthy diet (49.8%) and psychiatric or psychological therapy (36.4%) were also reported, reflecting recognition of the multidisciplinary nature of PD care. A small proportion of participants selected nonevidence‐based or nonclinical approaches such as nicotine/smoking (2.4%), caffeine consumption (6.2%), and alcohol use (1.7%) as potential means of symptom management, indicating variability in perceptions of disease management strategies. Additionally, 22.9% of respondents selected “love and hugs,” which may reflect a perception of the importance of emotional and social support in chronic disease care rather than a clinical treatment modality.

Overall, these findings suggest a reasonable level of awareness regarding established and supportive management strategies for PD, while also highlighting the persistence of misconceptions or nonclinical perceptions. These results underscore the need for improved public education on evidence‐based management approaches and clearer communication regarding validated therapeutic interventions.

### 3.7. Association Between Educational Level and Participants’ Knowledge and Awareness of PD

**FIGURE 1:**
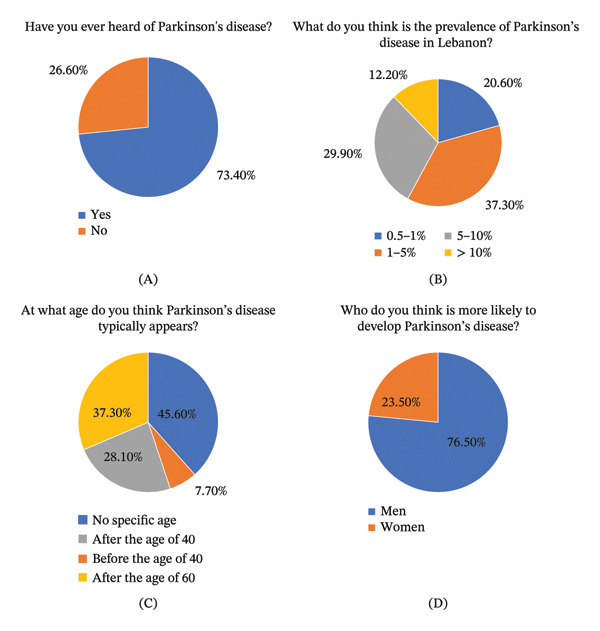
Pie chart illustrating the percentage of participants who had heard about Parkinson’s disease (A) and their knowledge regarding its frequency (B), possible age groups (C), and gender predominance (D).

Inferential statistics were conducted to examine the association between participants’ educational level and their awareness and perceptions of PD. The distribution of participants across educational levels is presented in Figure 1.

Table 8 shows a significant association between educational level and several indicators of PD awareness and perceptions. Participants with higher educational attainment were significantly more likely to have heard of PD, to have encountered a person with PD, and to express more informed perceptions regarding disease characteristics, including the belief that PD is not completely curable and the absence of a perceived relationship between COVID‐19 infection and PD (Table 8).

**TABLE 8:** Awareness and perceptions of Parkinson’s disease according to educational level.

Questions and answers	Intermediate education *n* (%)	Secondary education *n* (%)	Undergraduates *n* (%)	Master’s degree *n* (%)	PhD degree *n* (%)	Statistics (*p* value)
Have you ever heard about Parkinson’s disease (PD)?						< 0.001**^∗^**
Yes	8 (47.05%)	22 (42.30%)	245 (71.84%)	129 (87.16%)	23 (95.83%)	
No	9 (52.94%)	30 (57.69%)	96 (28.15%)	19 (12.83%)	1 (4.16%)	
Have you ever encountered a patient with PD?						< 0.001**^∗^**
Yes	4 (23.52%)	13 (25%)	125 (36.65%)	71 (47.97%)	16 (66.66%)	
No	13 (76.47%)	39 (75%)	216 (63.34%)	77 (52.02%)	8 (33.33%)	
Do you think the level of awareness regarding PD in Lebanon is sufficient?						0.153
Yes	1 (5.88%)	3 (5.76%)	6 (1.75%)	3 (2.02%)	0 (0%)	
No	16 (94.11%)	49 (94.23%)	335 (98.24%)	145 (97.97%)	24 (100%)	
What is your perception regarding the curability of Parkinson’s disease (PD)?						< 0.001**^∗^**
Yes (completely curable)	1 (5.88%)	5 (9.61%)	11 (3.22%)	7 (4.72%)	0 (0%)	
No (not completely curable)	6 (35.29%)	13 (25%)	173 (50.73%)	96 (64.86%)	20 (83.33%)	
I don’t know	10 (58.82%)	34 (65.38%)	157 (46.04%)	45 (30.40%)	4 (16.66%)	
Do you believe there is a relationship between COVID‐19 infection and Parkinson’s disease?						0.005**^∗^**
Yes	7 (41.17%)	22 (42.30%)	103 (30.20%)	39 (26.35%)	3 (12.50%)	
No	9 (52.94%)	29 (55.76%)	210 (61.58%)	95 (64.18%)	15 (62.50%)	
I don’t know	1 (5.88%)	1 (1.92%)	28 (8.21%)	14 (9.45%)	6 (25%)	
Total	17	52	341	148	24	

*Note:* Statistical analyses were conducted using the Kruskal–Wallis test. Bold values indicate statistically significant *p* values (*p* < 0.05). *p* values represent comparisons among educational level groups. A *p* value < 0.05 was considered statistically significant.

^∗^A statistically significant association.

Across all educational levels, the majority of participants perceived that the level of awareness regarding PD in Lebanon as insufficient (Table 8).

Table 9 examines the relationship between participants’ educational levels and their awareness and perceptions of general epidemiological aspects of PD. Statistically significant associations were observed between educational level and participants’ perceptions of the typical age of onset of PD (*p* = 0.003) as well as the gender distribution of the disease (*p* = 0.032).

**TABLE 9:** Association between educational level and awareness of epidemiological aspects of Parkinson’s disease.

Questions and answers	Intermediate education *n* (%)	Secondary education *n* (%)	Undergraduates *n* (%)	Master’s degree (%)	PhD degree *n* (%)	Statistics (*p* value)
What do you believe is the typical age of onset of Parkinson’s disease (PD)?						
No specific age	9 (52.94%)	32 (61.53%)	163 (47.80%)	49 (33.10%)	12 (50%)	0.003**^∗^**
< 40 years old	1 (5.88%)	7 (13.46%)	22 (6.45%)	14 (9.45%)	1 (4.16%)	0.367
> 40 years old	3 (17.64%)	11 (21.21%)	91 (26.68%)	53 (35.81%)	5 (20.83%)	0.113
> 60 years old^†^	4 (23.52%)	11 (21.21%)	132 (38.70%)	61 (41.21%)	10 (41.66%)	0.072
What is your perception of the frequency of Parkinson’s disease in Lebanon?						0.102
0.5%–1%	7 (41.17%)	10 (19.23%)	70 (20.52%)	26 (17.56%)	7 (29.16%)	
1%–5%	4 (23.52%)	17 (32.69%)	130 (38.12%)	57 (38.51%)	9 (37.50%)	
5%–10%^†^	1 (5.88%)	15 (28.84%)	102 (29.91%)	49 (33.10%)	7 (29.16%)	
> 10%	5 (29.41%)	10 (19.23%)	39 (11.43%)	16 (10.81%)	1 (4.16%)	
Who do you think is more likely to develop Parkinson’s disease?						0.032**^∗^**
Males^†^	11 (64.70%)	36 (69.23%)	259 (75.95%)	118 (79.72%)	21 (87.50%)	
Females	6 (35.29%)	16 (30.76%)	82 (24.04%)	30 (20.27%)	3 (12.50%)	
What is your perception of the contribution of genetic factors to Parkinson’s disease?						0.487
0%–10%	6 (35.29%)	13 (25%)	71 (20.82%)	38 (25.67%)	7 (29.16%)	
10%–15%^†^	6 (35.29%)	18 (34.61%)	140 (41.05%)	46 (31.08%)	7 (29.16%)	
50%–60%	4 (23.52%)	17 (32.69%)	99 (29.03%)	51 (34.45%)	9 (37.50%)	
> 90%	1 (5.88%)	4 (7.69%)	31 (9.09%)	13 (8.78%)	1 (4.16%)	
Total	17	52	341	148	24	

*Note:* Statistical analyses were performed using the Kruskal–Wallis test. Bold values indicate statistically significant *p* values (*p* < 0.05). The *p* values represent comparisons among educational level groups for each question. A *p* value < 0.05 was considered statistically significant.

^†^Items reflect participants’ perceptions and awareness of the epidemiological and genetic aspects of Parkinson’s disease based on the literature; responses were not classified as correct or incorrect.

^∗^A statistically significant association.

Participants with higher educational attainment (master’s and PhD degrees) were more likely to report that males are more frequently affected by PD, with 79.72% and 87.5%, respectively, selecting this option compared to 64.7% among participants with intermediate education.

Similarly, participants with advanced degrees more frequently reported that PD typically begins after the age of 60 years, with 41.21% of master’s degree holders and 41.66% of PhD holders selecting this response, compared with 23.5% of participants with intermediate education.

In contrast, no statistically significant associations were observed between educational level and perceptions regarding PD prevalence in Lebanon (*p* = 0.102) or the genetic contribution to PD (*p* = 0.487), suggesting relatively uniform awareness across educational groups for these aspects.

Overall, these findings suggest that higher educational attainment is associated with greater awareness of certain epidemiological features of PD, highlighting the need for targeted educational interventions, particularly among populations with lower educational attainment.

Table 10 presents participants’ responses concerning vitamin deficiencies and dietary patterns that have been reported or discussed in relation to PD, with multiple responses permitted. Across all educational levels, vitamin B12 was the most frequently selected item, while vitamin C was the least selected. Statistical analysis using the Kruskal–Wallis test revealed no significant association between educational level and the selection of any specific vitamin deficiency.

**TABLE 10:** Association between educational level and participants’ awareness of nutritional factors related to Parkinson’s disease.

Questions and answers	Intermediate education *n* (%)	Secondary education *n* (%)	Undergraduates *n* (%)	Master’s degree *n* (%)	PhD degree *n* (%)	Statistics (*p* value)
Which of the following vitamin deficiencies have been reported or discussed in relation to Parkinson’s disease?						
Vitamin B12^†^	10 (58.82%)	30 (57.69%)	228 (66.86%)	109 (73.64%)	18 (75%)	0.193
Vitamin B9^†^	3 (18.6%)	18 (34.61%)	131 (38.41%)	59 (39.86%)	7 (29.16%)	0.377
Vitamin C	1 (5.88%)	6 (11.53%)	41 (12.02%)	8 (5.40%)	(0%)	0.080
Vitamin D	6 (35.29%)	17 (32.69%)	132 (38.70%)	64 (43.24%)	9 (37.50%)	0.716
Which dietary patterns have been investigated in relation to Parkinson’s disease?						
Meat‐based^†^	5 (29.41%)	24 (46.15%)	157 (46.04%)	66 (44.59%)	9 (37.50%)	0.664
Vegetarian	5 (29.41%)	15 (28.84%)	114 (33.43%)	51 (34.45%)	7 (29.16%)	0.934
Plants and Meat‐based	6 (35.29%)	13 (25%)	69 (20.23%)	30 (20.27%)	4 (16.66%)	0.552
Dairy products^†^	5 (29.41%)	14 (26.92%)	76 (22.28%)	45 (30.40%)	6 (25%)	0.419
Total	17	52	341	148	24	

*Note:* The Kruskal–Wallis test was used for statistical analysis.

^†^Nutritional factors reported or investigated in previous studies in relation to Parkinson’s disease; however, evidence remains limited, inconsistent, or inconclusive for some associations.

Similarly, regarding dietary patterns, participants’ responses varied across educational levels. Participants with intermediate education most frequently selected a mixed meat‐ and plant‐based diet (35.3%), while those with higher educational attainment more commonly selected a meat‐based diet. However, the Kruskal–Wallis test indicated no statistically significant association between educational level and the selection of any dietary pattern.

Table 11 examines the association between educational level and participants’ awareness of factors reported or discussed in relation to PD. Statistically significant differences in responses were observed for specific items, particularly “influenza” (*p* < 0.001) and “low levels of dopamine” (*p* = 0.004).

**TABLE 11:** Association between educational level and recognition of factors associated with Parkinson’s disease.

Question and answers	Intermediate education *n* (%)	Secondary education *n* (%)	Undergraduates *n* (%)	Master’s degree *n* (%)	PhD degree *n* (%)	Statistics (*p* value)
Which of the following factors have been reported or discussed in relation to Parkinson’s disease (PD)?						
Influenza	7 (41.17%)	4 (7.69%)	20 (5.86%)	6 (4.05%)	0 (0%)	< 0.001**^∗^**
Smoking	6 (35.29%)	14 (26.92%)	59 (17.30%)	26 (17.56%)	3 (12.50%)	0.160
Low levels of dopamine^†^	3 (17.64%)	6 (11.53%)	126 (36.95%)	53 (35.81%)	9 (37.50%)	0.004**^∗^**
Getting old^†^	10 (58.82%)	24 (46.15%)	190 (55.71%)	95 (64.18%)	13 (54.16%)	0.204
Chronic disease^†^	5 (29.41%)	11 (21.21%)	72 (21.11%)	32 (21.62%)	4 (16.66%)	0.911
Genetic predisposition^†^	8 (47.05%)	31 (59.61%)	227 (66.56%)	107 (72.29%)	18 (75%)	0.139
Brain tumors	3 (17.64%)	9 (17.30%)	83 (24.34%)	41 (27.70%)	6 (25%)	0.607
Brain stroke	2 (11.76%)	7 (13.46%)	50 (14.66%)	32 (21.62%)	4 (16.66%)	0.368
Head trauma^†^	5 (29.41%)	12 (23.07%)	108 (31.67%)	44 (29.72%)	8 (33.33%)	0.789
Diabetes^†^	1 (5.88%)	3 (5.76%)	28 (8.21%)	8 (5.40%)	2 (8.33%)	0.830
Epilepsy^†^	5 (29.41%)	9 (17.30%)	64 (18.76%)	26 (17.56%)	2 (8.33%)	0.535
Vitamin deficiency^†^	9 (52.94%)	20 (38.46%)	164 (48.09%)	75 (50.67%)	13 (54.16%)	0.587
Mental pressure	5 (29.41%)	20 (38.46%)	86 (25.21%)	44 (29.72%)	6 (25%)	0.347
Sadness or depression	4 (23.52%)	18 (34.61%)	93 (27.27%)	38 (25.67%)	6 (25%)	0.776
Stress and fear	6 (35.29%)	20 (38.46%)	94 (27.56%)	43 (29.05%)	7 (29.16%)	0.575
Milk and dairy products^†^	3 (17.64%)	8 (15.38%)	34 (9.97%)	24 (16.21%)	4 (16.66%)	0.285
Total	17	52	341	148	24	

*Note:* Statistical analyses were performed using the Kruskal–Wallis test. Bold values indicate statistically significant *p* values (*p* < 0.05). The *p* values represent comparisons among educational level groups for each question. A *p* value < 0.05 was considered statistically significant.

^†^Factors previously reported as potentially associated with Parkinson’s disease; evidence for some associations remains limited or inconclusive.

^∗^A statistically significant association.

Participants with intermediate education were more likely to report influenza as being associated with PD (41.17%); however, this association is not supported by established scientific evidence, suggesting variability in participants’ understanding of potential PD‐related factors.

In contrast, recognition of dopamine‐related mechanisms increased with higher educational attainment, with PhD holders (37.5%) and undergraduates (36.95%) reporting higher awareness of this association. Other factors such as aging, chronic disease, genetic predisposition, and head trauma were frequently reported across all educational levels, with no statistically significant differences between groups.

Overall, these findings suggest a general level of awareness of commonly reported PD‐related factors across educational backgrounds, while also indicating that higher educational attainment may be associated with improved understanding of more specific neurobiological mechanisms. These results highlight the need for targeted educational strategies to improve public understanding of PD.

Participants demonstrated varying levels of awareness regarding the clinical manifestations of PD (Table 12), with educational background showing a significant association with symptom recognition. Reporting of cardinal motor symptoms—such as rest tremor, gait dysfunction, and walking difficulty—significantly increased with higher education levels. Rest tremor was reported by 52.9% of participants with intermediate education and by 95.9% of PhD holders (*p* = 0.001). Similarly, reporting of gait dysfunction increased from 41.2% among intermediate‐level participants to 91.7% among PhD holders (*p* < 0.001), while walking difficulty showed a comparable trend (47.1% vs. 75%, *p* = 0.017).

**TABLE 12:** Association between educational level and recognition of Parkinson’s disease symptoms.

Question and answers	Intermediate education *n* (%)	Secondary education *n* (%)	Undergraduates *n* (%)	Master’s degree *n* (%)	PhD degree *n* (%)	Statistics (*p* value)
Which of the following symptoms have been reported or are commonly associated with Parkinson’s disease (PD)?						
Rest tremor^†^	9 (52.94%)	38 (73.07%)	280 (82.11%)	130 (87.83%)	23 (95.83%)	0.001**^∗^**
Walking difficulty^†^	8 (47.05%)	22 (42.30%)	204 (59.82%)	97 (65.54%)	18 (75%)	0.017**^∗^**
Gait dysfunction^†^	7 (41.17%)	29 (55.76%)	239 (70.08%)	125 (84.45%)	22 (91.66%)	< 0.001**^∗^**
Dementia	4 (23.52%)	10 (19.23%)	95 (27.85%)	38 (25.67%)	5 (20.83%)	0.693
Anorexia	6 (35.29%)	9 (17.30%)	71 (20.82%)	21 (14.18%)	1 (4.16%)	0.050**^∗^**
Hypoxia	3 (17.64%)	12 (23.07%)	51 (14.95%)	23 (15.54%)	1 (4.16%)	0.312
Breathing difficulty	2 (11.76%)	8 (15.38%)	52 (15.24%)	20 (13.51%)	6 (25%)	0.685
Hypertension	4 (23.52%)	8 (15.38%)	46 (13.48%)	10 (6.75%)	1 (4.16%)	0.068
Muscle stiffness^†^	8 (47.05%)	20 (38.46%)	177 (51.90%)	77 (52.02%)	15 (62.50%)	0.307
Behavioral changes^†^	4 (23.52%)	11 (21.21%)	103 (30.20%)	49 (33.10%)	8 (33.33%)	0.544
Slow movement^†^	7 (41.17%)	31 (59.61%)	218 (63.92%)	97 (65.54%)	17 (70.83%)	0.304
Masked face^†^	6 (35.29%)	15 (28.84%)	108 (31.67%)	56 (37.48%)	11 (45.83%)	0.419
Constipation^†^	3 (17.64%)	16 (30.76%)	85 (24.92%)	28 (18.91%)	4 (16.66%)	0.332
Depression^†^	6 (35.29%)	19 (36.53%)	136 (39.88%)	62 (41.89%)	9 (37.50%)	0.952
Sleep disorders^†^	8 (47.05%)	18 (34.61%)	141 (41.34%)	65 (43.91%)	9 (37.50%)	0.782
Total	17	52	341	148	24	

*Note:* Statistical analyses were performed using the Kruskal–Wallis test. Bold values indicate statistically significant *p* values (*p* < 0.05). The *p* values represent comparisons among educational level groups for each question. A *p* value < 0.05 was considered statistically significant.

^†^Items reflect participants’ awareness of symptoms reported or commonly associated with Parkinson’s disease; responses are descriptive and were not classified as correct or incorrect answers.

^∗^A statistically significant association.

In contrast, anorexia was more frequently reported as a PD symptom among participants with lower educational levels (35.3% in intermediate education vs. 4.2% in PhD holders; *p* = 0.050). No statistically significant associations were observed for other symptoms, including depression, constipation, bradykinesia, or sleep disturbances.

Overall, these findings suggest that educational attainment is associated with differences in awareness of PD clinical manifestations, highlighting the need for targeted educational interventions to improve symptom recognition across all educational levels.

Table 13 evaluates the association between educational attainment and participants’ awareness of strategies used or perceived to manage PD symptoms. Statistically significant associations were observed for responses related to medicine (*p* = 0.012), DBS (*p* = 0.028), and physiotherapy (*p* < 0.001).

**TABLE 13:** Association between educational level and awareness of strategies for managing Parkinson’s disease symptoms.

Questions and answers	Intermediate education *n* (%)	Secondary education *n* (%)	Undergraduates *n* (%)	Master’s degree *n* (%)	PhD degree *n* (%)	Statistics (*p* value)
Which of the following approaches are used or perceived to help manage Parkinson’s disease (PD) symptoms?						
Medicine^†^	15 (88.23%)	32 (61.53%)	275 (80.64%)	123 (83.10%)	20 (83.33%)	0.012**^∗^**
Deep brain stimulation^†^	4 (23.52%)	6 (11.53%)	51 (14.95%)	37 (25%)	7 (29.16%)	0.028**^∗^**
Exercise^†^	8 (47.05%)	36 (69.23%)	183 (53.66%)	85 (57.43%)	14 (58.33%)	0.271
Nicotine/smoking	1 (5.88%)	1 (1.92%)	7 (2.05%)	5 (3.37%)	(0%)	0.682
Alcohol consumption	(0%)	(0%)	7 (2.05%)	3 (2.02%)	(0%)	0.748
Physiotherapy^†^	6 (35.29%)	20 (38.46%)	170 (49.85%)	78 (52.70%)	22 (91.66%)	< 0.001**^∗^**
Psychiatric/psychological therapy	7 (41.17%)	17 (32.69%)	126 (36.95%)	52 (35.13%)	10 (41.66%)	0.923
Healthy diet	7 (41.17%)	28 (53.84%)	165 (48.38%)	76 (51.35%)	14 (58.33%)	0.774
Vitamin Supplements^†^	6 (35.29%)	23 (44.23%)	201 (58.94%)	86 (58.10%)	13 (54.16%)	0.120
Social support	3 (17.64%)	18 (34.61%)	68 (19.94%)	37 (25%)	7 (29.16%)	0.141
Caffeine consumption	1 (5.88%)	2 (3.84%)	20 (5.86%)	11 (7.43%)	2 (8.33%)	0.888
Total	17	52	341	148	24	

*Note:* Statistical analyses were performed using the Kruskal–Wallis test. Bold values indicate statistically significant *p* values (*p* < 0.05). The *p* values represent comparisons among educational level groups for each question. A *p* value < 0.05 was considered statistically significant.

^†^Items reflect participants’ awareness of approaches reported or perceived to help manage Parkinson’s disease symptoms; responses do not necessarily reflect evidence‐based effectiveness and were not classified as correct or incorrect.

^∗^A statistically significant association.

Medicine was the most frequently reported management strategy across all educational levels, with high levels of awareness among participants with intermediate education (88.23%), master’s degrees (83.10%), and PhDs (83.33%). DBS awareness increased with educational level, ranging from 11.53% among secondary education participants to 29.16% among PhD holders, suggesting greater familiarity with advanced treatment options among more highly educated individuals. Similarly, physiotherapy awareness was highest among PhD participants (91.66%), indicating increased recognition of rehabilitative approaches with higher educational attainment.

In contrast, nonmedical or nonconventional approaches such as nicotine, alcohol, and “love and hugs” were reported at low frequencies across all educational levels, with no statistically significant differences observed between groups.

Overall, these findings suggest that higher educational attainment is associated with greater awareness of a broader range of PD management strategies, including both pharmacological and rehabilitative approaches.

### 3.8. Association Between Occupational Sector and Participants’ Knowledge and Awareness of PD

**FIGURE 2:**
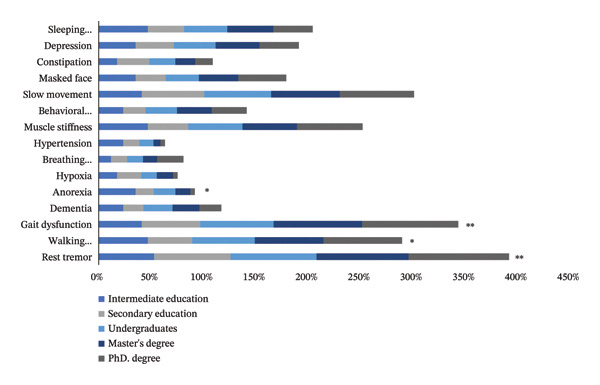
Stacked bar chart illustrating the distribution of Parkinson’s disease symptom recognition among participants across different educational levels. Note: The Kruskal–Wallis test was used for statistical analysis. ^∗^Refers to significant association (*p* < 0.05); ^∗∗^highly significant association (*p* < 0.01).

Inferential statistical analyses were performed among employed participants to examine the association between the occupational sector and participants’ awareness of PD. Among the 287 employed respondents, 67 (23.3%) were employed in the healthcare sector, whereas the majority, 220 participants (76.7%), worked in non‐healthcare fields. Figure 2 illustrates the distribution of participants according to occupational sector.

Table 14 demonstrates a significant association between occupational sector and awareness regarding PD. Participants working in the healthcare sector were significantly more likely to have heard of PD (*p* < 0.001) and to have encountered a patient with PD (*p* < 0.001) compared with participants from non‐healthcare sectors.

**TABLE 14:** Association between occupational sector and participants’ awareness of Parkinson’s disease.

Questions and answers	Healthcare sector *n* (%)	Non‐healthcare sectors *n* (%)	Statistics (*p* value)
Have you ever heard about Parkinson’s disease (PD)?			
Yes	64 (95.52%)	163 (74.09%)	< 0.001**^∗^**
No	3 (4.47%)	57 (25.90%)	
Have you ever encountered a patient with PD?			
Yes	47 (70.14%)	94 (42.72%)	< 0.001**^∗^**
No	20 (29.85%)	126 (57.27%)	
Do you think the level of awareness regarding PD in Lebanon is sufficient?			
Yes	1 (1.49%)	4 (1.81%)	1
No	66 (98.50%)	216 (98.18%)	
What is your perception regarding the curability of Parkinson’s disease (PD)?			
Yes (completely curable)	1 (1.49%)	6 (2.72%)	0.188
No (not completely curable)	48 (71.64%)	131 (59.54%)	
I don’t know	18 (26.86%)	83 (37.72%)	
Do you believe there is a relationship between COVID‐19 infection and Parkinson’s disease?			
Yes	18 (26.86%)	67 (30.45%)	0.335
No	42 (62.68%)	141 (64.09%)	
I don’t know	7 (10.44%)	12 (5.45%)	
Total	67	220	

*Note:* Statistical analyses were performed using the chi‐square test. Bold values indicate statistically significant associations (*p* < 0.05).

^∗^A statistically significant association.

Almost all participants, regardless of occupational sector, reported that awareness of PD in Lebanon is insufficient, with more than 98% in both groups expressing this view. Although a higher proportion of healthcare workers reported that PD cannot be completely cured (71.64% vs. 59.54%), the difference was not statistically significant (*p* = 0.188), and a considerable proportion of participants in both groups selected “I don’t know.” No statistically significant association was observed regarding participants′ responses to the question related to COVID‐19 and PD.

Overall, these findings suggest that working in the healthcare sector is associated with greater awareness and exposure to PD; however, differences between occupational groups were less evident for some disease‐related perceptions and beliefs.

Table 15 examines the association between occupational sector and participants’ awareness of general information related to PD, comparing responses between healthcare and non‐healthcare professionals. A significantly higher proportion of healthcare workers selected “> 60 years old” as the typical age of onset of PD compared with participants from non‐healthcare sectors (61.19% vs. 38.63%; *p* = 0.001). In addition, fewer healthcare professionals selected “no specific age” (26.86% vs. 41.81%; *p* = 0.031), suggesting greater awareness of the age‐related characteristics of PD.

**TABLE 15:** Association between occupational sector and awareness of epidemiological aspects of Parkinson’s disease.

Questions and answers	Healthcare sector *n* (%)	Non‐healthcare sectors *n* (%)	Statistics (*p* value)
What do you believe is the typical age of onset of Parkinson’s disease (PD)?			
No specific age	18 (26.86%)	92 (41.81%)	0.031**^∗^**
< 40 years old	3 (4.47%)	18 (8.18%)	0.425
> 40 years old	22 (32.83%)	60 (27.27%)	0.440
> 60 years old^†^	41 (61.19%)	85 (38.63%)	0.001**^∗^**
What is your perception of the frequency of Parkinson’s disease in Lebanon?			
0.5%–1%	9 (13.43%)	49 (22.27%)	0.053
1%–5%	25 (37.33%)	79 (35.90%)	
5%–10%^†^	18 (26.86%)	68 (30.90%)	
> 10%	15 (22.38%)	24 (10.90%)	
Who do you think is more likely to develop Parkinson’s disease?			
Men^†^	59 (88.05%)	173 (78.63%)	0.110
Women	8 (11.94%)	47 (21.36)	
What is your perception of the contribution of genetic factors to Parkinson’s disease?			
0%–10%	11 (16.41%)	58 (26.36%)	0.296
10%–15%^†^	30 (44.77%)	83 (37.72%)	
50%–60%	21 (31.34%)	61 (27.72%)	
> 90%	5 (7.46%)	18 (8.18%)	
Total	67	220	

*Note:* The chi‐square test was used to evaluate associations between occupational sector and responses regarding the perceived age of onset and gender susceptibility of Parkinson’s disease (PD). Fisher’s exact test was applied to the “< 40 years” response category because of small expected cell frequencies. The Kruskal–Wallis test was used to assess associations between occupational sector and participants’ perceptions of PD prevalence in Lebanon and the contribution of genetic factors to PD development. Bold values indicate statistically significant associations (*p* < 0.05).

^†^Items reflect participants’ perceptions and awareness of the epidemiological and genetic aspects of PD based on the literature; responses were not classified as correct or incorrect.

^∗^A statistically significant association.

No statistically significant associations were observed regarding participants’ perceptions of PD prevalence in Lebanon, genetic contribution to disease development, or sex‐related susceptibility (*p* > 0.05). Nevertheless, a higher proportion of healthcare participants selected the 10%–15% range for genetic contribution and identified men as being more likely to develop PD (88.05% vs. 78.63%).

Overall, these findings suggest that participants working in the healthcare sector demonstrated greater awareness regarding the typical age of onset of PD; however, awareness of other epidemiological aspects of the disease appeared relatively comparable across occupational groups.

Regarding the question “Which of the following vitamin deficiencies do you believe are associated with PD?,” participants were allowed to select multiple responses, and the results are presented in Table 16.

**TABLE 16:** Association between occupational sector and participants’ awareness of nutritional factors related to Parkinson’s disease.

Questions and answers	Healthcare sector *n* (%)	Non‐healthcare sector *n* (%)	Statistics (*p* value)
Which of the following vitamin deficiencies have been reported or discussed in relation to Parkinson’s disease?			
Vitamin B12^†^	43 (64.17%)	163 (74.09%)	0.123
Vitamin B9^†^	28 (41.79%)	76 (34.54%)	0.311
Vitamin C	8 (11.94%)	13 (5.90%)	0.110
Vitamin D	27 (40.29%)	81 (36.81%)	0.666
Which dietary patterns have been investigated in relation to Parkinson’s disease?			
Meat‐based^†^	29 (43.28%)	100 (45.45%)	0.781
Vegetarian	28 (41.79%)	55 (25%)	0.009**^∗^**
Plant and meat‐based	11 (16.41%)	48 (21.81%)	0.391
Dairy products^†^	18 (26.86%)	62 (28.18%)	0.878
Total	67	220	

*Note:* Statistical analyses were performed using the chi‐square test. Fisher’s exact test was applied to the “Vitamin C” response category because of small expected cell frequencies. Bold values indicate statistically significant associations (*p* < 0.05).

^†^Nutritional factors that have been reported or investigated in relation to Parkinson’s disease; however, evidence for some associations remains limited, inconsistent, or inconclusive.

^∗^A statistically significant association.

Table 16 examines the association between occupational sector and participants’ awareness of nutritional factors related to PD. Most comparisons between healthcare and non‐healthcare participants did not demonstrate statistically significant differences. However, a significant association was observed regarding the selection of a vegetarian diet, with healthcare participants choosing this option more frequently than non‐healthcare participants (41.79% vs. 25%; *p* = 0.009).

Awareness regarding vitamin deficiencies potentially associated with PD showed no statistically significant differences between occupational groups (*p* > 0.05). Vitamin B12 was the most frequently selected option in both groups, followed by vitamin B9. Responses related to vitamin C and vitamin D also did not differ significantly between healthcare and non‐healthcare participants.

Overall, the findings suggest moderate awareness of nutrition‐related factors associated with PD across both occupational sectors, with generally comparable response patterns between healthcare and non‐healthcare participants.

Table 17 evaluates the association between occupational sector and participants’ awareness of factors associated with PD. Healthcare professionals more frequently selected low dopamine levels (71.64% vs. 24.09%, *p* < 0.001) and aging (71.64% vs. 57.27%, *p* = 0.045) compared with participants from non‐healthcare sectors, indicating greater awareness of key biological and age‐related aspects of PD.

**TABLE 17:** Association between occupational sector and recognition of factors associated with Parkinson’s disease.

Question and answers	Healthcare sector *n* (%)	Non‐healthcare sectors *n* (%)	Statistics (*p* value)
Which of the following factors have been reported or discussed in relation to Parkinson’s disease (PD)?			
Influenza	5 (7.46%)	10 (4.54%)	0.353
Smoking	13 (19.40%)	30 (13.63%)	0.328
Low levels of dopamine^†^	48 (71.64%)	53 (24.09%)	< 0.001**^∗^**
Getting old^†^	48 (71.64%)	126 (57.27%)	0.045**^∗^**
Chronic disease^†^	12 (17.91%)	45 (20.45%)	0.729
Genetic predisposition^†^	51 (76.11%)	141 (64.09%)	0.076
Brain tumors	16 (23.88%)	47 (21.36%)	0.736
Brain stroke	11 (16.41%)	36 (16.36%)	1
Head trauma^†^	19 (28.35%)	68 (30.90%)	0.762
Diabetes^†^	8 (11.94%)	12 (5.45%)	0.096
Epilepsy^†^	11 (16.41%)	32 (14.54%)	0.845
Vitamin deficiency^†^	40 (59.70%)	104 (47.27%)	0.094
Mental pressure	15 (22.38%)	66 (30%)	0.278
Sadness or depression	17 (25.37%)	69 (31.38%)	0.875
Stress and fear	20 (29.85%)	64 (29.09%)	1
Milk and dairy products^†^	9 (13.43%)	27 (12.27%)	0.834
**Total**	67	220	

*Note:* Statistical analyses were performed using the chi‐square test. Fisher’s exact test was applied to the “Influenza” and “Diabetes“ response categories because of small expected cell frequencies. Bold values indicate statistically significant associations (*p* < 0.05).

^†^Factors reported in previous studies as potentially associated with Parkinson’s disease; however, evidence for some associations remains limited or inconclusive.

^∗^A statistically significant association.

For most other factors, including genetic predisposition, head trauma, vitamin deficiencies, and chronic disease, no statistically significant differences were observed between occupational groups, although selection rates were generally higher among healthcare participants. In addition, participants from both sectors identified factors such as mental pressure, depression, stress, and fear, reflecting variability in perceptions regarding factors potentially associated with PD.

Overall, these findings suggest that healthcare professionals demonstrated greater awareness of certain biological and age‐related characteristics of PD; however, awareness patterns across both occupational sectors remained variable regarding several other factors associated with the disease.

Table 18 assesses the association between occupational sector and participants’ awareness of PD symptoms. Healthcare professionals demonstrated higher levels of recognition of several motor and nonmotor manifestations of PD compared with participants from non‐healthcare sectors. Statistically significant differences were observed for walking difficulty (76.11% vs. 59.54%, *p* = 0.014), gait dysfunction (89.55% vs. 72.72%, *p* = 0.005), muscle stiffness (62.68% vs. 46.36%, *p* = 0.025), slow movement (86.56% vs. 59.54%, *p* < 0.001), and masked face (47.76% vs. 30.90%, *p* = 0.013).

**TABLE 18:** Association between occupational sector and recognition of Parkinson’s disease symptoms.

Question and answers	Healthcare sector *n* (%)	Non‐healthcare sectors *n* (%)	Statistics (*p* value)
Which of the following symptoms have been reported or are commonly associated with Parkinson’s disease (PD)?			
Rest tremor^†^	59 (88.05%)	185 (84.09%)	0.447
Walking difficulty^†^	51 (76.11%)	131 (59.54%)	0.014**^∗^**
Gait dysfunction^†^	60 (89.55%)	160 (72.72%)	0.005**^∗^**
Dementia	23 (34.32%)	62 (28.18%)	0.361
Anorexia	19 (28.35%)	29 (13.18%)	0.005**^∗^**
Hypoxia	8 (11.94%)	41 (18.63%)	0.266
Breathing difficulty	16 (23.88%)	27 (12.27%)	0.030**^∗^**
Hypertension	12 (17.91%)	16 (7.27%)	0.017**^∗^**
Muscle stiffness^†^	42 (62.68%)	102 (46.36%)	0.025**^∗^**
Behavioral changes^†^	28 (41.79%)	57 (25.90%)	0.015**^∗^**
Slow movement^†^	58 (86.56%)	131 (59.54%)	< 0.001**^∗^**
Masked face^†^	32 (47.76%)	68 (30.90%)	0.013**^∗^**
Constipation^†^	29 (43.28%)	34 (15.45%)	< 0.001**^∗^**
Depression^†^	45 (67.16%)	79 (36.90%)	< 0.001**^∗^**
Sleep disorders^†^	40 (59.70%)	82 (37.27%)	0.001**^∗^**
Total	67	220	

*Note:* Statistical analyses were performed using the chi‐square test. Bold values indicate statistically significant associations (*p* < 0.05).

^†^Items reflect participants’ awareness of symptoms reported or commonly associated with Parkinson’s disease; responses are descriptive and were not classified as correct or incorrect.

^∗^A statistically significant association.

Healthcare participants also more frequently selected nonmotor symptoms associated with PD, including constipation (43.28% vs. 15.45%, *p* < 0.001), depression (67.16% vs. 36.90%, *p* < 0.001), sleep disorders (59.70% vs. 37.27%, *p* = 0.001), and behavioral changes (41.79% vs. 25.90%, *p* = 0.015).

In addition, some symptoms not commonly associated with PD, such as anorexia, breathing difficulties, and hypertension, were selected by participants from both occupational groups, with higher selection frequencies observed among healthcare workers.

Overall, participants working in the healthcare sector demonstrated greater awareness of both motor and nonmotor symptoms of PD, although variability in symptom attribution remained evident across both occupational sectors.

The data in Table 19 reveal that participants working in the healthcare sector demonstrated significantly greater awareness of medication as a method to reduce or manage the symptoms of PD compared with those in non‐healthcare sectors (95.52% vs. 75.90%, *p* < 0.001). This finding suggests that professional exposure to medical knowledge may be associated with better awareness of evidence‐based PD management strategies.

**TABLE 19:** Association between occupational sector and awareness of strategies for managing Parkinson’s disease symptoms.

Question and answers	Healthcare sector *n* (%)	Non‐healthcare sectors *n* (%)	Statistics (*p* value)
Which of the following approaches are used or perceived to help manage Parkinson’s disease symptoms?			
Medicine^†^	64 (95.52%)	167 (75.90%)	< 0.001**^∗^**
Deep brain stimulation^†^	17 (25.37%)	36 (16.36%)	0.107
Exercise^†^	38 (56.71%)	123 (55.90%)	1
Nicotine/smoking	2 (2.98%)	4 (1.81%)	0.627
Alcohol consumption	2 (2.98%)	5 (2.27%)	0.667
Physiotherapy^†^	41 (61.19%)	110 (50%)	0.125
Psychiatric/psychological therapy	29 (43.28%)	83 (37.72%)	0.475
Healthy food	39 (58.20%)	102 (46.36%)	0.096
Vitamin supplements^†^	45 (67.16%)	124 (56.36%)	0.122
Social support	18 (26.86%)	58 (26.36%)	1
Caffeine consumption	7 (10.44%)	9 (4.09%)	0.065
Total	67	220	

*Note:* Statistical analyses were performed using the chi‐square test. Fisher’s exact test was applied to the “Nicotine,” “Alcohol,” and “Caffeine” response categories because of small expected cell frequencies. Bold values indicate statistically significant associations (*p* < 0.05).

^†^Items reflect participants’ awareness of approaches reported or perceived to help manage Parkinson’s disease symptoms; responses do not necessarily reflect evidence‐based effectiveness and were not classified as correct or incorrect.

^∗^A statistically significant association.

Although awareness of other scientifically supported approaches, such as DBS, physiotherapy, exercise, healthy diet, and vitamin supplementation, was generally higher among healthcare participants, these differences were not statistically significant. In addition, non‐evidence‐based options such as nicotine, alcohol, and caffeine were infrequently selected in both groups, with no significant differences observed.

Overall, these results indicate that while healthcare professionals show greater awareness of pharmacological management of PD, knowledge of broader multidisciplinary management strategies remains relatively similar across occupational sectors, highlighting the need for improved public education on comprehensive PD care.

## 4. Discussion

The findings of our study reveal a moderate level of awareness about PD among the Lebanese population, with 73.4% having heard of the disease (Figure 1A). However, only 39.3% reported having met a patient with PD, and misconceptions were still being observed, as 43% of participants were uncertain whether PD can be completely cured. In addition, only 29.90% were aware of the estimated frequency of PD in Lebanon (5%–10%) (Figure 1B). Regarding the age of onset, nearly half of the participants indicated that PD does not have a specific age of onset, whereas 37.30% reported that PD commonly occurs after the age of 60 years (Figure 1C). Previous studies have shown that late‐onset PD affects approximately 2%‐3% of individuals older than 65 years [12], whereas early‐onset PD accounts for about 5%‐6% of cases and generally occurs before the age of 50 years, although some sources define it as occurring before the age of 40 years [13]. These findings suggest variability in participants’ knowledge regarding the epidemiology and age of onset of PD. The observed uncertainty may reflect limited public awareness of PD and the clinical heterogeneity of the disease.

Around three‐quarters of the participants indicated that men are more likely to be affected by PD (Figure 1D). Previous studies have reported that the prevalence of PD in men is approximately 1.5 to 2 times higher than that in women [14]. Some studies have proposed that hormonal differences, including the possible neuroprotective role of estrogen, may contribute to this difference [15]. These findings suggest that a considerable proportion of the Lebanese population is aware of the higher occurrence of PD among men.

More than half of the participants identified genetic factors and aging as factors associated with PD. According to the literature, brain sensitivity increases with age while repair mechanisms and cellular processes decline, making aging one of the most significant risk factors for PD development [16]. Regarding genetic contributions, fewer than half of the participants selected the 10%–15% range. To date, more than 90 genetic loci have been identified as potential risk factors for PD [17]. The findings of the present study suggest moderate public awareness regarding the association between aging, genetic factors, and PD, although knowledge of the magnitude of genetic contribution appears to be variable.

The analysis of participants’ responses regarding nutritional factors associated with PD revealed variability in awareness. A substantial proportion of respondents (67.9%) identified vitamin B12 deficiency as being linked to PD, suggesting some awareness of its neurological relevance. However, only 37.5% selected vitamin B9 deficiency, while others selected vitamin D (39.2%) and vitamin C (9.6%), reflecting possible uncertainty regarding the specific roles of vitamins in PD. Some studies have reported a potential association between vitamin C deficiency and PD, given its role in brain function and oxidative stress regulation; however, the available evidence remains limited and inconclusive [18]. As for vitamin D, it is important for brain development and the maturation of brain activity and is associated with several neurological disorders, including PD. A higher prevalence of vitamin D deficiency has been observed among individuals with PD compared with control populations [19]. Furthermore, a study conducted in Lebanon found that the mean vitamin D level was significantly lower in PD patients than in the control group [20]. Similarly, both vitamin B9 and vitamin B12 appear to have protective roles, as they are involved in the homocysteine (Hcy) metabolism cycle, contributing to the maintenance of low levels of the neurotoxic Hcy in the blood [21, 22]. The analysis of our study highlights a mix of accurate knowledge and significant misconceptions among participants regarding the nutritional factors associated with PD. While awareness of vitamin B12’s role was relatively high, uncertainty surrounding other vitamins, particularly B9, D, and C, was also observed. These findings suggest variability in participants’ understanding of the potential relationship between nutritional factors and neurological health. Further public health education may help improve community awareness regarding nutrition‐related aspects of PD.

Similarly, misconceptions regarding dietary factors associated with PD were observed among participants. While 44.8% correctly identified a meat‐based diet as potentially associated with PD, 33% selected a vegetarian diet. In addition, 25.1% of respondents considered a diet rich in dairy products to be potentially associated, which is consistent with some studies reporting a relationship between dairy intake and PD risk [23, 26]. Previous research has also suggested that plant‐based dietary patterns may be associated with potential benefits in PD, whereas diets high in animal fat or cholesterol have been linked to an increased risk of PD [24, 25]. Overall, these findings indicate variability in participants’ understanding of dietary factors associated with PD and suggest the need for clearer public health education regarding nutrition and PD.

In addition, 18.60% of respondents identified smoking as a potential risk factor associated with PD. These findings suggest varying levels of awareness among participants regarding the relationship between smoking and PD. Previous studies have reported an inverse association between nicotine exposure and the risk of PD. In 2015, a study published by the *American Journal of Epidemiology* reported that people with a history of smoking had a 45% lower risk of developing PD [27]. In a study published in 2012, researchers at the University of Washington in Seattle observed that individuals exposed to second‐hand smoke, even if they had never smoked themselves, exhibited a lower risk level similar to that of observed among active smokers [27].

Interestingly, researchers have observed a potential link between COVID‐19 and the onset of PD, although the exact underlying mechanisms remain unclear [28–30]. However, a growing body of literature, including a recent comprehensive review by Boura et al. [31], has further investigated this association. Similar to other systemic infections, COVID‐19 has been implicated in both transient and long‐term worsening of PD symptoms, particularly in individuals experiencing long COVID‐19. Moreover, emerging clinical reports suggest a possible increase in new‐onset PD cases following SARS‐CoV‐2 infection, though this connection remains speculative. Mechanistically, SARS‐CoV‐2 is known to provoke a systemic inflammatory response and, in severe cases, a cytokine storm [32]. This response may alter the blood–brain barrier [33], permitting the infiltration of inflammatory mediators or the virus itself into the central nervous system (CNS). This can trigger a neuroinflammatory cascade, oxidative stress, and neurodegeneration [34], especially in midbrain dopaminergic neurons, central to PD pathophysiology [35]. A meta‐analysis evaluating the impact of viral and bacterial infections on PD risk indicated that infected individuals had a 20% higher risk of developing PD compared to controls [36]. Supporting this, Baizabal‐Carvallo and Alonso‐Juarez [37] noted that viral infections have long been implicated in the multifactorial pathogenesis of PD. For instance, Beckers and his colleagues [38] reported cases where patients developed motor symptoms of PD shortly after COVID‐19, with previous nonmotor symptoms. Furthermore, experimental research has demonstrated that SARS‐CoV‐2 proteins may increase α‐synuclein expression and aggregation, facilitating Lewy body formation in vitro [39, 40]. This finding supports the theory that COVID‐19 could contribute to neurodegeneration and synucleinopathies, particularly in individuals with genetic or environmental susceptibility. Additional mechanisms have also been proposed by Outeiro and Krisko [41], suggesting that COVID‐19–related inflammation may accelerate biological aging, potentially contributing to the earlier onset of PD. Moreover, SARS‐CoV‐2 may impair dopamine synthesis by downregulating ACE2 receptors, thereby reducing the expression of aromatic L‐DOPA decarboxylase (DDC), a key enzyme in dopamine production [42]. Although the causal relationship between COVID‐19 and PD remains unconfirmed, the accumulation of clinical and experimental findings points toward a biologically plausible connection that merits continued investigation. In this context, our study assessed public awareness regarding the potential association between COVID‐19 and PD. Notably, 67% of respondents believed that COVID‐19 could be associated with an increased burden of PD. In contrast, only 6.40% recognized influenza infection as a possible factor associated with PD, suggesting lower awareness of the potential relationship between infectious diseases and PD pathogenesis.

Furthermore, 21.2% of the participants identified chronic diseases as a factor associated with PD, while 18.2% selected epilepsy. However, only 7.2% recognized diabetes as a possible contributor to PD. These findings suggest variability in participants’ awareness regarding medical conditions that have been investigated as potential factors associated with PD. Research indicates that diabetes mellitus and insulin resistance increase both the risk of developing PD and its progression [43]. A 2016 meta‐analysis of seven cohort studies involving over 1.7 million individuals concluded that diabetic patients have approximately a 38% higher risk of developing PD [43]. Additionally, studies have shown that individuals with epilepsy are 2.5 times more likely to develop PD compared to those without epilepsy [44].

A total of 24.4%, 16.4%, and 30.3% of participants believed that PD is associated with brain tumors, stroke, and head trauma, respectively. These findings indicate variability in participants’ understanding of neurological conditions and factors that may be associated with PD. A meta‐analysis found that the association between stroke and PD remains unclear; however, the two conditions may share common pathophysiological mechanisms and preventive treatment. Another meta‐analysis reported that a history of head trauma is associated with an increased risk of developing PD [45]. Conversely, research indicates that Parkinsonism resulting from brain tumors is relatively uncommon [46].

On the other hand, approximately 27%–30% of participants believed that mental pressure, sadness or depression, stress, and fear are associated with an increased risk of PD. These findings suggest differing perceptions among participants regarding the potential relationship between psychological factors and PD. A study found that stress can trigger PD by causing neuronal degeneration [47]. Also, some features of stress, such as shaking from fear, resemble PD symptoms. Moreover, depression was found to be an early sign of PD in 2.5% of patients, with 25% developing these symptoms within the first 2 years [47]. The results reveal a limited understanding among participants regarding medically established and emerging risk factors for PD.

While some participants identified chronic diseases, epilepsy, and head trauma as factors associated with PD, awareness of the reported association between diabetes and PD was relatively low. Additionally, misconceptions were observed regarding factors such as brain tumors and emotional stress, for which the scientific evidence remains limited or inconclusive. These findings underscore the need for improved public education that differentiates between evidence‐based risk factors and common myths, thereby enhancing early recognition, prevention, and management of PD.

Since PD is characterized by the death of dopaminergic neurons and reduced dopamine levels in the brain [1], only 33.7% of the participants in this study were able to identify this response. Similarly, a survey conducted in Central Uganda assessing knowledge of PD revealed that nearly half of the respondents were unaware of the part of the body system affected by the disease [9]. The findings highlight a significant gap in participants’ understanding of the fundamental pathology of PD, with only about one‐third identifying low dopamine levels as its primary mechanism. In summary, the results suggest that the most commonly recognized factors associated with PD among the Lebanese population are aging, genetics, COVID‐19, a meat‐based diet, and vitamin B12 deficiency, with more than 50% of participants selecting these factors. In contrast, 18%–40% of participants identified low dopamine levels, chronic diseases, head trauma, epilepsy, vitamin B9 and D deficiencies, and psychological factors such as mental pressure, sadness, depression, stress, and fear as being associated with PD. Additionally, fewer than 20% of participants associated PD with influenza, brain stroke, brain tumors, vitamin C deficiency, diabetes, or milk and dairy consumption. Approximately 33% of participants associated a vegetarian diet with PD risk. These responses reflect variability in participants’ understanding of factors reported in the literature to be related to PD [48]. Moreover, 24% of participants identified brain tumors and 18.6% identified smoking as factors associated with PD. Meanwhile, further research is needed to clarify the potential associations between PD and factors such as COVID‐19, influenza, dietary patterns, psychological stress, and stroke, and to better determine their clinical and epidemiological significance.

In our survey, between 60% and 83% of participants were able to identify key motor symptoms of PD, including resting tremors, gait dysfunction, difficulty walking, and bradykinesia. Additionally, 51% identified muscle stiffness as a symptom. These signs have been classified as cardinal symptoms of PD in previous studies [49]. However, awareness of nonmotor symptoms was lower, with only 34%–42% of participants identifying masked face, depression, and sleep disturbances. In contrast, prior research has emphasized that these nonmotor symptoms are not only common but also essential for the diagnosis and management of PD [49, 50]. This comparison highlights a disparity between public awareness and established clinical knowledge, underlining the need for broader education on both motor and nonmotor manifestations of the disease. Furthermore, a study conducted at the Lebanese American University (LAU) in Lebanon investigated the association between PD and depression, finding that 46% of the PD patients enrolled were diagnosed with depression [51]. The study included 200 patients over the age of 35, representing 10 different personality types. In our survey, fewer than 25% of participants identified constipation [52], behavioral changes, dementia [50], breathing difficulties [53], and hypertension [54] as symptoms of PD. While hypotension is more commonly observed in PD patients, dysfunction of the autonomic nervous system can cause both neurogenic OH and supine hypertension. Moreover, medications used to treat OH can exacerbate supine hypertension [54], highlighting the importance of further research into these symptoms. Additionally, 16.7% of participants identified hypoxia as a symptom of PD, although it is more appropriately classified in the literature as a potential risk factor [55]. Furthermore, 18.6% identified anorexia as a symptom of PD. While PD patients may experience a reduced appetite due to factors such as depression, nausea, and loss of smell, anorexia itself is not classified as a direct symptom of PD [56]. These findings point to the need for improved public education on the broader symptomatology and risk factors associated with PD.

Several studies have investigated public awareness of PD symptoms across different populations. For example, a cross‐sectional survey conducted among Xhosa‐speaking Black South Africans found that only 18% of participants were able to identify PD based on its symptoms [57]. Similarly, a study involving 1258 individuals from a multiethnic urban Asian population utilized the Knowledge of PD Questionnaire (KPDQ) and found that tremor was the most frequently recognized symptom, identified by 79% of respondents. Overall, motor symptoms were more commonly recognized than nonmotor symptoms [58]. In Saudi Arabia, a large‐scale online survey distributed via Twitter collected 2609 valid responses out of 3050 participants. In that study, tremor was the most recognized symptom (86.1%), and 56% of respondents identified imbalance as a symptom. Recognition of motor symptoms ranged from 31.3% to 86.1%, whereas awareness of non‐motor symptoms remained low, ranging between 4.1% and 24% [59]. In comparison, our survey of the Lebanese population demonstrated a relatively comparable or even slightly higher level of awareness of some motor symptoms. Overall, recognition of motor symptoms in our study closely aligns with the figures reported in the Saudi and Asian studies. Nonmotor symptoms were less frequently identified but still showed slightly better recognition than that observed in the Saudi population. These comparative findings indicate that while basic awareness of PD’s cardinal motor symptoms is fairly strong in Lebanon, public understanding of nonmotor symptoms remains insufficient, highlighting a shared international need for more comprehensive and targeted education efforts.

More than half of the participants in our study reported that there are no effective treatments for PD. In contrast, 4.2% believed that PD could be completely cured, while approximately 43% were uncertain. As PD is a progressive neurodegenerative disorder, where motor symptoms typically emerge after 70%–80% of dopaminergic neurons have already degenerated, it is currently considered incurable [59]. However, various medical interventions and therapies are available to help manage symptoms and reduce their severity. The finding that over half of the participants reported PD as incurable may reflect general awareness of the serious and chronic nature of the disease. Nonetheless, the high percentage of respondents who were unsure indicates a significant gap in public understanding of the disease, highlighting the need for enhanced education regarding disease characteristics and available treatment options.

Approximately 80% of the participants in our survey indicated that medications help manage the symptoms of PD. This reflects a good level of awareness regarding pharmacological treatment. Medications such as dopamine agonists are important for symptom management, as they mimic dopamine by binding to dopaminergic postsynaptic receptors and activating signaling pathways similar to those normally triggered by dopamine [60, 61]. In contrast, only 17.9% of participants identified DBS surgery as a treatment option for PD. DBS is typically recommended for patients whose symptoms cannot be adequately controlled with medication alone [61]. The procedure enhances neuronal stimulation, promoting neuroplasticity and neurogenesis, which in turn may improve motor symptoms and reduce the required dosage of levodopa, thereby minimizing medication‐related side effects [59].

Our findings suggest that while the Lebanese population has a reasonable understanding of the role of medication in PD management, there is a significant lack of awareness regarding the benefits and application of DBS surgery, despite the presence of specialized DBS centers in Lebanon. This lack of awareness is not unique to Lebanon. For instance, a study conducted at a single center in India on knowledge, attitudes, and perceptions of DBS in PD revealed substantial knowledge gaps and widespread misconceptions among both patients and caregivers [62]. These findings emphasize the need for targeted educational campaigns to improve public and patient understanding of advanced treatment options like DBS.

Between 50% and 55% of participants in our survey selected physiotherapy, vitamin supplements, regular exercise, and healthy eating as strategies for managing PD symptoms. Regarding physiotherapy, a meta‐analysis involving approximately 8000 participants demonstrated the effectiveness of various physiotherapy interventions in improving motor symptoms and overall functional ability among individuals with PD [63]. Similarly, physical exercise has been reported to be associated with improvements in both motor and nonmotor symptoms of PD, including motor performance, mood, and behavioral outcomes [64].

In terms of nutritional support, several supplements, such as omega‐3 fatty acids, whey protein, and coenzyme Q10, have been reported to help alleviate PD symptoms [65–67]. Additionally, a case study involving patients with PD and vitamin C deficiency reported symptom improvement following increased vitamin C intake [18]. While there is evidence of an association between vitamin D deficiency and the severity of PD symptoms, current research does not yet confirm vitamin D as an effective treatment for symptom management [19].

Although an awareness level of 50%–55% among participants is notable, likely reflecting the general understanding that a healthy lifestyle contributes to overall well‐being, the fact that nearly half of the respondents did not identify these nonpharmacological management strategies highlights a gap in public knowledge. This underscores the need to increase awareness regarding the potential role of exercise, physiotherapy, and nutrition in supporting the quality of life of individuals with PD.

Regarding beverages, only 6.2% of respondents in our survey believed that caffeine‐containing beverages may be associated with the management of PD symptoms. While caffeine may not directly reduce symptoms, research suggests it may slow the progression of PD, which still represents a potential therapeutic benefit for patients [68]. Additionally, around 2% of participants believed that alcohol or cigarette consumption might alleviate PD symptoms. However, studies indicate that alcohol may exacerbate certain PD symptoms, particularly sleep disturbances such as insomnia, and may also interfere with the effectiveness of PD medications. Therefore, alcohol consumption is generally discouraged in individuals with PD [69].

Interestingly, although cigarette smoking is known for its harmful effects, research has identified nicotine as a potentially promising therapeutic molecule for PD. Studies have shown that nicotine may help slow disease progression and improve symptoms by influencing dopaminergic pathways [70–72]. Moreover, experimental studies using the *Drosophila melanogaster* model have demonstrated that nicotine, a cholinergic agonist, can increase dopamine levels and support the survival of dopaminergic neurons [73].

Our results suggest that participants were generally aware of the adverse health effects of smoking and alcohol consumption; however, their knowledge regarding the potential associations between nicotine, caffeine, and PD was limited. This gap in understanding may be related to the fact that over 85% of respondents did not work in the healthcare sector, and approximately 30% held a master’s or PhD degree. Approximately 23% of participants in our survey believed that social support may be associated with improvement in PD symptoms. Hugs are known to stimulate the release of beneficial hormones such as dopamine and oxytocin, which promote relaxation and reduce feelings of depression. In this way, physical affection may serve as a natural stimulus for dopamine release [74]. However, while these associations are generally beneficial for mental health, there is currently insufficient scientific evidence to confirm that affection or emotional support significantly improves the clinical symptoms of PD. Nonetheless, the relatively low percentage of participants who selected this option may reflect a broader lack of awareness about the psychological and neurological benefits of emotional support, suggesting a need for further research in this area. In addition, 36.5% of participants believed that psychiatric therapy may be associated with the management of PD symptoms. While psychiatric therapy does not address the motor symptoms of PD, it plays a vital role in managing the nonmotor symptoms such as depression, anxiety, and fear, which commonly accompany the disease [75]. It is unclear from the responses whether participants considered psychiatric therapy as supportive care for the psychological aspects of PD, or whether they viewed it as a treatment for the motor symptoms of the disease. The relatively high percentage may also reflect a broader misunderstanding of PD as a mental health condition rather than a neurodegenerative movement disorder. This response may reflect limited or inaccurate perceptions regarding neurological disorders, where conditions such as epilepsy and PD are sometimes associated with cognitive impairment or mental instability [76]. These findings highlight the need for improved public education and scientifically grounded awareness regarding the nature of PD, particularly in Lebanon, where misconceptions were observed among participants from the general population, as well as academic and professional backgrounds.

Our results revealed a statistically significant association between educational level and respondents’ awareness of PD (*p* < 0.001), as well as having met a person with PD (*p* < 0.001). Higher educational levels were associated with a greater likelihood of having heard of PD or reporting prior contact with someone with the condition. This finding may reflect differences in exposure to health‐related information. In Lebanon, PD is sometimes referred to colloquially as the “tremor disease,” which may contribute to variations in public recognition and understanding of the condition. Additionally, there was a significant association between educational level and the belief about whether PD can be treated (*p* < 0.001). Individuals with higher education levels were more likely to report that PD cannot be treated, and none of the respondents holding a PhD selected alternative responses. A similar trend was observed in responses regarding the typical age of PD onset (*p* = 0.003). Undergraduate students were more likely to select “no specific age,” possibly due to a lack of knowledge. In contrast, the majority of respondents with master’s or PhD degrees selected “after the age of 60,” suggesting a greater familiarity with the disease.

Furthermore, a significant association was found between educational level and the perception that men are at a higher risk of developing PD (*p* = 0.032). The perception that men are more susceptible to PD was more frequently reported among participants with higher educational levels. A similar pattern emerged in responses concerning COVID‐19 and its possible link with PD. A higher educational level was associated with responses indicating no perceived relationship between COVID‐19 and PD. This may be due to the fact that, despite being highly educated, these individuals do not necessarily have a background in healthcare or medicine.

Regarding risk factors, educational level was significantly associated with the selection of “influenza” and “low levels of dopamine” as potential contributors to PD. Differences were observed in response distributions across educational groups. Participants with intermediate education (41.17%) more frequently selected “influenza,” whereas those with undergraduate (36.95%), master’s (35.81%), and doctoral (PhD) (37.50%) degrees more frequently identified “low levels of dopamine.”

In terms of symptoms, educational level was significantly associated with the recognition of “rest tremor,” “walking difficulty,” “gait dysfunction,” and “anorexia,” with variation in responses across educational levels (Figure 2).

When considering methods of symptom management, a significant association was found between educational level and the selection of “medication,” “DBS,” and “physiotherapy,” with graduate participants being more likely to choose these options than undergraduates.

Overall, these findings suggest that educational level is significantly associated with awareness and knowledge of key aspects of PD. This is consistent with the results of a cross‐sectional study conducted in Thailand among 125 patients, which identified education level as a significant predictor of PD knowledge [77].

Our findings demonstrated a strong and statistically significant association between employment in the healthcare sector and both awareness of PD and prior interaction with PD patients. Notably, participants from non‐healthcare sectors were significantly more likely to select “no specific age” as the onset of PD, suggesting a gap in knowledge regarding disease onset. In contrast, healthcare professionals more accurately selected “after the age of 60,” which aligns with clinical understanding of PD onset. Interestingly, a significantly higher proportion of healthcare workers identified a vegetarian diet as a factor possibly correlated with PD, although this is not supported by current scientific evidence. This may reflect a general awareness of the nutritional role of meat in brain health, but it also underscores the need for more targeted education about evidence‐based PD risk factors, even among healthcare professionals.

In terms of risk factors, participants in the healthcare sector were significantly more likely to identify “low levels of dopamine” and “aging” as factors associated with PD. Regarding symptoms, working in the healthcare sector was significantly correlated with the identification of several PD‐related symptoms, including walking difficulty, gait dysfunction, muscle stiffness, slow movement, masked facial expressions, constipation, depression, and sleep disturbances.

**FIGURE 3:**
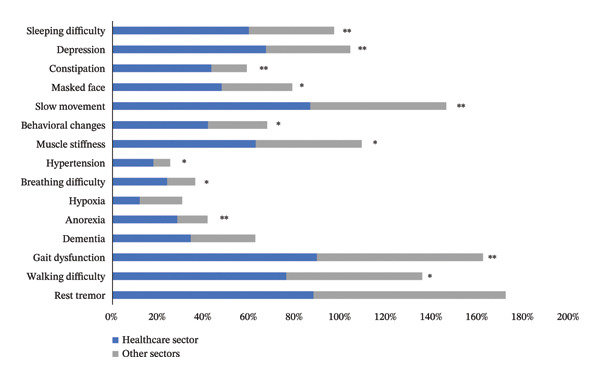
Stacked bar chart illustrating the distribution of Parkinson’s disease symptom recognition among participants across different occupational sectors. Note: The chi‐square test was used for statistical analysis. ^∗^Refers to significant association (*p* < 0.05); ^∗∗^highly significant association (*p* < 0.01).

However, a considerable proportion of healthcare professionals also identified symptoms such as anorexia (28.4%), breathing difficulty (23.9%), and hypertension (17.9%), which are not typically described in association with PD in the literature (Figure 3). This finding suggests variability in knowledge regarding the clinical presentation of PD among healthcare sector participants and highlights the need for further education and training on the topic.

When it comes to treatment and management, the only significant association found between occupational sector and treatment options was in choosing “medication,” which is the most commonly used method recommended by physicians. These findings suggest a correlation between working in the healthcare sector and higher awareness of many key aspects of PD, although some misconceptions were also observed.

Furthermore, it is important to highlight that there are currently no specialized medical centers for PD in Lebanon. The only dedicated facility is the PD and Movement Disorders Unit at Notre Dame des Secours Hospital in Byblos, which focuses on PD, other movement disorders, and DBS surgery. This lack of specialized centers has contributed to a shortage of well‐trained nurses knowledgeable in PD care. As a result, even when patients are supported by nurses at home or in hospitals, those caregivers may lack the necessary training to provide appropriate care. Although healthcare professionals demonstrated greater awareness of PD compared to participants from other sectors, their responses in this study still revealed notable gaps in knowledge and awareness. While previous studies have explored public knowledge of PD [34, 78] and its association with educational level [53], none have directly examined the relationship between occupational sectors and PD awareness. Given that certain professions typically require higher levels of education, occupation may be associated with differences in awareness and understanding of the disease. To the best of our knowledge, this is the first study to specifically investigate the association between occupational background and awareness of PD. By addressing this gap, our research offers novel insights into how professional roles may shape knowledge of PD. Further studies focusing on this correlation are warranted to enhance understanding of the sociodemographic, educational, and occupational factors associated with public awareness of PD.

The clinical importance of these findings lies in the identification of gaps in public awareness regarding PD, particularly in relation to its risk factors and clinical presentation. Limited knowledge may contribute to delayed recognition of symptoms and postponement of medical consultation, which can ultimately affect patient outcomes. Understanding sociodemographic and occupational differences in awareness may assist healthcare professionals and policymakers in designing targeted educational interventions to improve early recognition and support timely referral and management. Future nationwide studies involving more diverse and representative populations may further clarify these associations and help guide the development of targeted public health awareness and educational strategies related to PD.

## 5. Conclusion

This study represents the first comprehensive evaluation of PD awareness within the Lebanese population, encompassing public knowledge, educational attainment, and diverse occupational sectors. Our results demonstrate that although general awareness of PD exists and is notably higher among individuals with advanced educational attainment and those working in the healthcare sector, significant misconceptions persist across all groups. These misunderstandings are particularly related to the disease’s etiology, risk factors, nonmotor symptoms, and treatment options, indicating that even higher educational levels and professional exposure do not fully eliminate gaps in accurate knowledge.

Notably, widespread misconceptions were identified, including erroneous beliefs regarding dietary habits and the misattribution of symptoms such as anorexia and hypertension to PD. Additionally, there was limited awareness of scientifically supported risk factors, such as dopamine deficiency, diabetes, and head trauma. Awareness of the neurological implications of COVID‐19 varied among respondents, and a considerable proportion reported a perceived association between the virus and PD, although current scientific evidence remains inconclusive. These insights highlight the urgent need to address both knowledge deficits and misinformation within the general population.

The broader implications of these findings are significant. A limited understanding of PD’s clinical presentation and risk profile may impede early recognition, timely diagnosis, and appropriate management. Furthermore, the scarcity of specialized PD care centers in Lebanon, coupled with inadequate training among nonspecialist healthcare providers and caregivers, further compounds the challenges faced by patients and their families. Addressing these gaps is essential to improving PD outcomes and supporting the needs of affected individuals across the country.

Furthermore, interventional studies exploring targeted educational campaigns and healthcare‐based awareness programs are warranted to generate evidence on effective approaches for enhancing public knowledge, facilitating early recognition, and improving disease management among individuals with PD.

## 6. Limitations and Strengths

The use of a convenience self‐selection sampling method through online social media platforms may have introduced selection bias and limited the generalizability of the findings to the broader Lebanese population. The use of an online self‐administered questionnaire may introduce selection bias and limit generalizability due to the exclusion of individuals without internet access or digital literacy and may be subject to self‐report bias. While this method allowed for efficient data collection, it introduced several limitations. First, the online nature of the survey made it difficult to reach a broad and diverse segment of the population. Only 582 individuals participated, and the sample was heavily skewed by gender, with 81% of participants being female. In addition, the study population was predominantly composed of younger, well‐educated, and technologically engaged individuals, which may not accurately reflect the demographic distribution of the Lebanese population. This imbalance may be due to several factors, including greater responsiveness among females and younger individuals to online health‐related surveys, as well as differences in internet access, digital literacy, and survey engagement across age groups and educational levels. Women are often more involved in caregiving roles and may be more interested in health topics such as PD, which could have increased their likelihood of participating. Additionally, the reliance on digital literacy excluded some potential participants, particularly older adults or individuals with limited access to or familiarity with online tools. Some respondents may have had difficulty using Google Forms or may have chosen not to participate due to technological barriers. Therefore, the findings should be interpreted with caution and may not be fully representative of the entire Lebanese population.

Furthermore, we could not confirm whether participants answered based on actual knowledge, guesses, or personal preferences. For instance, 76% of respondents indicated that men are more likely to develop PD. Given that the majority of respondents were female, it is unclear whether this response was based on accurate knowledge or influenced by assumptions or wishful thinking. These factors further limit the generalizability of the findings and should be considered when interpreting the results.

On the other hand, another limitation of this study is that several complex biomedical concepts related to PD were simplified into closed‐ended questionnaire items to ensure clarity and feasibility in a large population‐based survey. While this approach allowed efficient data collection and quantitative analysis, it may have introduced a degree of oversimplification of multifactorial disease mechanisms. In particular, some items included factors with varying or inconclusive levels of scientific evidence, which may have influenced participants’ responses based on perception rather than established clinical knowledge.

In addition, the use of predefined response categories does not allow exploration of participants’ reasoning or the depth of understanding behind their choices. Therefore, the findings should be interpreted as reflecting participants’ awareness and perceptions rather than objective medical knowledge. This may limit direct comparison with clinically validated knowledge assessments.

Despite these limitations, this study has several strengths. It is among the first to assess public awareness and misconceptions about PD in the Lebanese population. The sample size exceeded the minimum required for statistical significance, enhancing the reliability of the findings. The survey included participants from various regions, age groups, and educational and occupational backgrounds, allowing for a broader analysis of knowledge disparities. Furthermore, the questionnaire underwent expert validation and pilot testing, ensuring its relevance, clarity, and comprehensiveness. This robust methodological framework supports the validity of the insights gained and provides a strong foundation for future public health interventions in Lebanon.

## Author Contributions

Jeanne d’arc Bacha was responsible for the conceptualization of the project. The methodology was developed by Jeanne d’arc Bacha, Noura El‐Loubani, Hanan Hijazi, Sarah Mawed, and Layal El Bayda. Software development was carried out by Noura El‐Loubani, Sarah Mawed, and Layal El Bayda. Validation was performed by Ziad Fajloun, Rabih Roufayel, Hervé Kovacic, Hanan Hijazi, and Jeanne d’arc Bacha. The investigation was conducted by Ziad Fajloun and Jeanne d’arc Bacha. The original draft was written by Noura El‐Loubani, and the review and editing were done by Ziad Fajloun, Hervé Kovacic, and Jeanne d’arc Bacha. Supervision and project administration were both handled by Jeanne d’arc Bacha.

## Funding

This research received no external funding.

## Disclosure

All authors have read and agreed to the published version of the manuscript.

## Conflicts of Interest

The authors declare no conflicts of interest.

## Supporting Information

Additional supporting information can be found online in the Supporting Information section.

## Supporting information


**Supporting Information** Supporting File S1: Questionnaire used for the assessment of knowledge and misconceptions about Parkinson’s disease.

## Data Availability

The data that support the findings of this study are available from the corresponding author upon reasonable request.
