# Supplementary material for: Knowledge and Misconceptions About Parkinson’s Disease in Lebanon: A Cross‐Sectional Survey
Source: Parkinsons Dis. 2026 Jul 8;2026:1010419. doi: 10.1155/padi/1010419 (PMC13343311; doi:10.1155/padi/1010419)
Supplement: Supplementary file 1 — Supporting Information Supporting File S1: Questionnaire used for the assessment of knowledge and misconceptions about Parkinson’s disease. [file PADI-2026-1010419-s001.docx]

**Supplementary** **File 1**

The Google Form survey used in this study to assess knowledge and awareness of Parkinson’s disease (PD) in the Lebanese population is presented below. In this questionnaire, (*) indicates mandatory questions, (○) indicates that only one response is allowed, and (□) indicates that multiple responses are permitted.

**Study information, objectives, and consent**

This study aims to assess the level of awareness, knowledge, and perceptions regarding Parkinson’s disease (PD) among the Lebanese population. Specifically, it evaluates participants’ knowledge of PD, including symptoms, risk factors, perceived causes, and management strategies, as well as sociodemographic factors associated with PD awareness.

Participation in this study is entirely voluntary.

I confirm that I have read and understood the information about this study. I voluntarily agree to participate. My responses are anonymous and will be used for research purposes only. I understand that I may withdraw from the survey at any time before submission.

o Yes, I agree to participate
o No (end survey)

**Eligibility criteria**

**Are you 15 years or older?**o Yes
o No (end survey)

**Are you currently living in Lebanon?**o Yes
o No (end survey)

**Confidentiality statement**

This survey is anonymous. Please do not enter your name or email address.

1. **Age** *

Your answer

1. **Gender** *

- Male
- Female

1. **Educational level** *

- Intermediate education
- Secondary education
- Undergraduate
- Master’s degree
- PhD degree

1. **Current job** *

- No job
- Education sector
- Employee
- Nurse
- Doctor
- First aid crew
- Healthcare sector

1. **Have you ever heard of Parkinson’s disease (PD)?***

- Yes
- No

1. **Do you suffer from Parkinson’s disease (PD)?** *

- Yes
- No

1. **Do you have a PD family member?** *

- Yes
- No
- I don't know

1. **Have you ever met a PD patient?***

- Yes
- No

1. **Do you think there is enough public awareness about PD in Lebanon?** *

- Yes
- No

1. **In your opinion, what is the frequency of PD in Lebanon?** *

- 0.5 - 1%
- 1 - 5%
- 5 - 10%
- >10%

1. **Do you think that PD can be treated completely?***

- Yes
- No
- I don't know

1. **In your opinion, at what age does PD appear?***

- There is no specific age
- Before the age of 40
- After the age of 40
- After the age of 60

1. **In your opinion, between men and women, who is more likely to develop PD?** *

- Men
- Women

1. **In your opinion, how much effect do genetics have on developing PD?** *

- 0%-10%
- 10%-15%
- 50%-60%
- 90%

1. **In your opinion, which of the following Vitamins' deficiencies lead to PD?** *

- Vitamin B12
- Vitamin B9
- Vitamin D
- Vitamin C

1. **In your opinion, which diet is more likely to develop PD?** *

- Plants and a meat-based diet
- Vegetarian
- Meat-based diet
- Diet rich in dairy products

1. **Do you think that COVID-19 virus may cause Parkinson’s disease?** *

- Yes
- No
- I don't know

1. **Choose the symptoms of Parkinson’s disease.***

- Rest tremor
- Walking difficulty
- Gait dysfunction
- Dementia
- Anorexia
- Hypoxia
- Breathing difficulty
- Hypertension
- Muscle stiffness
- Behavioral changes
- Slow movement
- Masked face
- Constipation
- Depression
- Sleeping difficulty

1. **Choose the factors that may cause Parkinson’s disease.** *

- Influenza
- Smoking
- Low level of dopamine
- Getting old
- Chronic diseases
- Genes
- Brain tumors
- Brain stroke
- Head trauma
- Diabetes
- Epilepsy
- Vitamin B12 deficiency
- Mental pressure
- Sadness or depression
- Stress and fear
- Milk and dairy products

1. **Choose the ways to manage or reduce the symptoms of Parkinson’s disease.** *

- Medicine
- Deep-brain stimulation surgery
- Exercise
- Nicotine/smoking
- Alcohol consumption
- Physiotherapy
- Psychiatric/psychological therapy
- Healthy diet
- Vitamin supplements
- Social support
- Caffeine consumption
